# CAR-T cells and BiTEs in solid tumors: challenges and perspectives

**DOI:** 10.1186/s13045-021-01067-5

**Published:** 2021-04-19

**Authors:** Julien Edeline, Roch Houot, Aurélien Marabelle, Marion Alcantara

**Affiliations:** 1 Medical Oncology, Centre Eugène Marquis, University of Rennes 1, Rennes, France; 2 Department of Hematology, CHU Rennes, INSERM U1236, University of Rennes, Rennes, France; 3 Département d’Innovation Thérapeutique et d’Essais Précoces (DITEP), INSERM U1015, INSERM CIC1428, Université Paris Saclay, Gustave Roussy, France; 4 Center for Cancer Immunotherapy, INSERM U932, Institut Curie, PSL Research University, Paris, France

**Keywords:** CAR-T cells, BiTEs, Bispecific antibodies, Solid tumors

## Abstract

Chimeric antigen receptor (CAR)-modified T cells and BiTEs are both immunotherapies which redirect T cell specificity against a tumor-specific antigen through the use of antibody fragments. They demonstrated remarkable efficacy in B cell hematologic malignancies, thus paving the way for their development in solid tumors. Nonetheless, the use of such new drugs to treat solid tumors is not straightforward. So far, the results from early phase clinical trials are not as impressive as expected but many improvements are under way. In this review we present an overview of the clinical development of CAR-T cells and BiTEs targeting the main antigens expressed by solid tumors. We emphasize the most frequent hurdles encountered by either CAR-T cells or BiTEs, or both, and summarize the strategies that have been proposed to overcome these obstacles.

## Background

The recent years have seen the revolution of immunotherapy enter the clinic. This new class of agents uses different therapeutic approaches, most of which focus are based on T cells. While immune checkpoint inhibitors (ICI) have been approved in a wide range of solid tumors, other immunotherapies such as chimeric antigen receptor (CAR)-T cells and T cell redirecting bispecific T cell Engager (BiTE) have exclusively been approved in hematologic malignancies and are yet poorly studied in solid tumors [1–3]. Immune therapies which have been approved in hematologic malignancies target “ideal” antigens, namely CD19, CD20 and BCMA, for several reasons: these antigens are present on all tumors cells; the normal cells which also express these antigens are dispensable and can be eliminated without excessive “on-target, off-tumor” toxicity; these antigens are expressed on the surface and as such are easily accessible without the need for presentation through the major histocompatibility complex (MHC). Because of impressive results observed with BiTEs and CAR-T cells in hematologic malignancies, many researchers, both academic and industrial, are trying to expand these therapies to the field of solid tumors. In this review, we present the specificities related to the development of CAR-T cells and BiTEs in solid tumors, illustrate the main challenges encountered in this development, and highlight approaches to overcome these obstacles.

## CAR-T cells and BiTEs: overview of their development in solid tumors

CAR-T cells and BiTEs’ mechanisms of action rely on redirecting T cell specificity against a tumor antigen through the use of antibody fragments. CAR-T cells are genetically engineered T cells (either autologous or allogeneic) that express a chimeric antigen receptor (CAR). Indeed, the CAR is composed of an extracellular single-chain variable fragment (scFv), “antibody-like” antigen-binding domain, which recognizes a tumor-specific antigen in a MHC independent manner, and intracellular signaling domains, which mimic T cell receptor (TCR) activation [4]. Adoptive cell therapies (ACT) also encompass ex vivo expansion and infusion of tumor-infiltrating lymphocytes (TILs) [5] and genetic redirection of non-therapeutic endogenous lymphocytes with a TCR which recognizes a tumor-specific antigen presented through the MHC [6]. BiTEs are recombinant proteins made of two scFv from two different antibodies, one targeting a tumor-specific antigen and the other targeting the effector T cell (mostly CD3). Thus, endogenous T cells are recruited at the tumor site and redirected to kill cancer cells in vivo [7].

Main characteristics and differences between CAR-T cells and BiTEs are summarized in Table 1. For both types of therapies, the first critical step is the selection of a tumor-specific antigen. The most frequently targeted antigens currently used for the development of these therapies in solid tumors are summarized in Table 2.

**Table 1:** Comparison of the main characteristics of CAR-T cells and BiTEs

	CAR-T cells	BiTEs
Effector cell	Ex vivo engineered T cells	Unmanipulated T cells
Personalized	Yes (at least for autologous CAR-T cells)	No
Availability	Delayed (weeks, for autologous CAR-T cells)	Immediate (“off-the-shelf”)
Logistics	+ + + (leukapheresis, transportation, genetic engineering, conservation…)	+
Half-life	Long (weeks-months)	Short
Dosing	Single infusion (“one shot”)	Repeat dosing
Efficacy	Long-lasting (immunological memory)	Suspensive
Administration	Requires lymphodepleting chemotherapy prior to CAR-T infusion	Requires multiple injections or continuous infusion over several months
Chronic toxicity	Possible	No
Cost	+ + +	+ +

**Table 2:** Most frequently targeted antigens, and clinical examples of application

	Expression in normal tissue	Expression in solid tumors	CAR-T examples	BiTEs examples	References
HER2	Low expression in normal tissue	Overexpression in selected solid tumors	Responses seen in biliary tract cancer, rhabdomyosarcoma, mostly partial; 1 toxic death reported	14 patients treated, 1 response	[8, 73, 74, 101, 102]
EGFRvIII	No expression in normal tissue	Specific mutation present in some glioblastoma	18 glioblastoma patients: median PFS of 1.3 months, 1 outlier 1 long responder after intraventricular injection Acquired resistance after 1 injection through antigen loss No toxicity	1 response in 8 evaluable patients	[17, 81, 103, 104]
Mesothelin	Low expression in normal tissue	Expression in some solid tumors	18 mesothelioma patients treated in combination with anti-PD1: 2 complete responses and 5 partial responses No toxicity	50 treated patients, no response	[83, 105]
GD2	Very limited expression in normal tissue	Constant in neuroblastoma	6 patients treated, some responses in 3 patients. No toxicity	Only preclinical	[106]
Glypican-3	Low expression in normal tissue	Frequent expression in hepatocellular carcinoma, expression in selected tumors	13 HCC patients treated, 2 partial responses 1 grade 5 CRS	Phase 1 ongoing	[31, 107]
CEA (CEACAM5)	Low expression in normal tissue	Expression in multiple solid tumors	10 patients treated: 2 partial metabolic responses No severe toxicity	2 responders among 11 patients treated in combination with anti-PD-L1	[88, 108]
PSMA	Low expression in normal tissue	Frequent expression in prostate	2 of 5 patients had a partial response. No toxicity	3 responders among 15 patients treated in a dose-escalation trial	[33, 34]
Claudin 18.2	Low expression in normal gastric tissue	Expression in some solid tumors	4 partial responses among 10 patients treated. 3 grade 2 on-target gastric toxicities	Phase 1 ongoing	[37, 39]
EpCAM	Low expression in normal tissue	Expression in multiple solid tumors	Only preclinical	Catumoximab: Demonstrated efficacy of intraperitoneal injection to control the symptoms of peritoneal carcinomatosis Severe gastrointestinal toxicity precluding further development of this drug	[12, 109]
AFP (intracellular)	Low expression in normal tissue	Expression in hepatocellular carcinoma and some other tumors	Partial responses described with 2 different TCR-engineered T cells targeting AFP	Not applicable	[51, 110]

One of the major limitations of antigen selection in solid tumors is that some low-level expression is often found in normal tissue exposing the patient to a risk of “on-target, off-tumor” toxicity. This was for example the explanation for one fatal case of early development of HER2-targeting CAR-T cells, where HER2 expression on normal lung epithelial cells was deemed responsible for cytokine release [8]. Furthermore, some antigens are restricted to some tumor types, while other have a broader spectrum across various tumors. Thus, solid tumors often require prior screening to ensure that the target is expressed.

Overall, both BiTEs and CAR-T cells have shown evidence of activity in solid tumors, but these results still need to be improved before entering clinical practice. Current evidence does not support one strategy over the other in solid tumors.

## Current challenges with T cell directed therapies in solid tumors

**Fig. 1:**
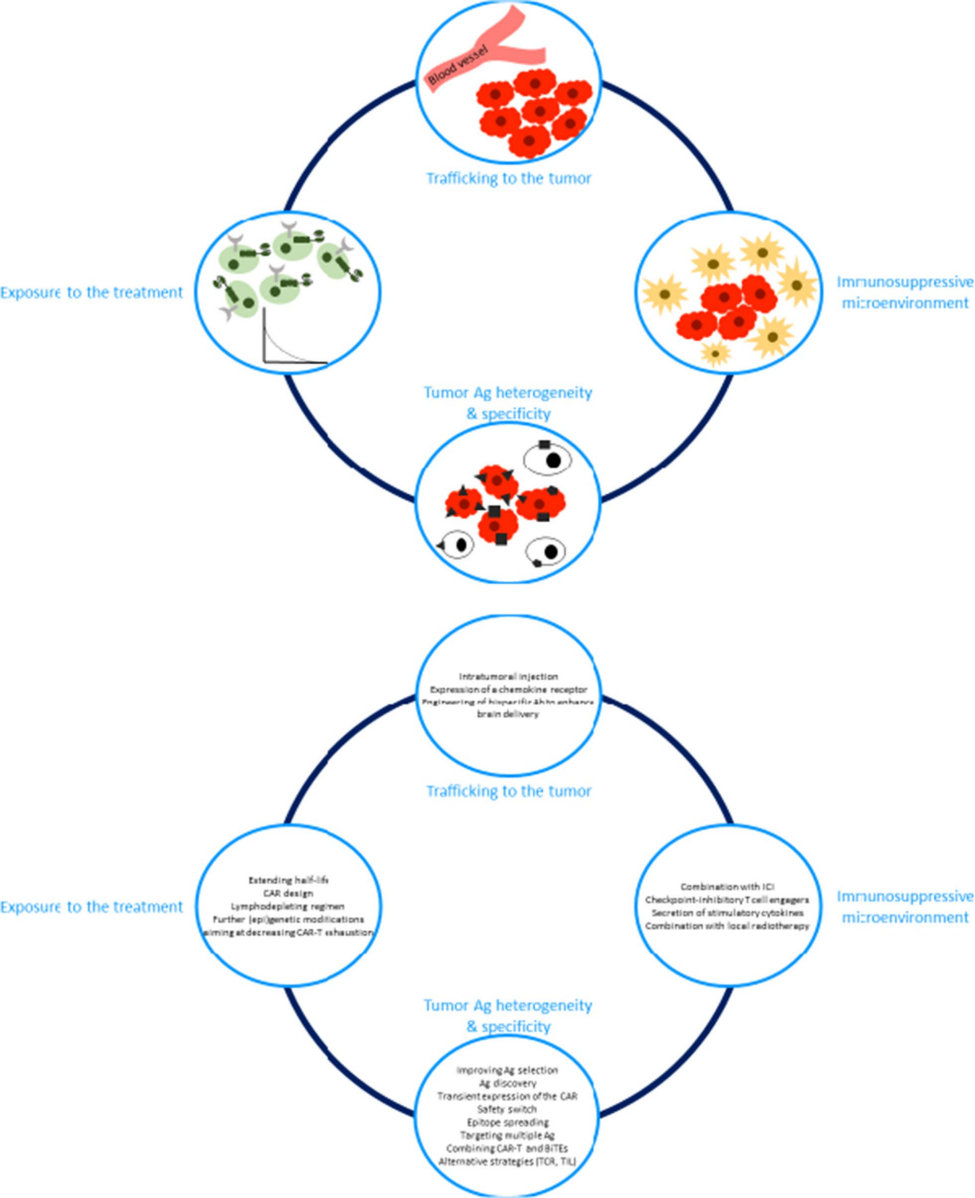
Schematic representation of **a** the challenges encountered by CAR-T cells and BiTEs for their development in solid tumors and **b** some solutions proposed to overcome these challenges. Ag: antigen, Ab: antibody, ICI: immune checkpoint inhibitors

A recent systematic review compared the results of CAR-T cells in hematologic versus solid tumors [9]. The pooled response rate was 71% in hematological malignancies *versus* 29% in solid tumors. This review might still overestimate the efficacy because some negative trials may not be reported. However, this review confirms that these therapies might be efficient in solid tumors, although responses seem to be more difficult to achieve compared to hematologic malignancies. The different challenges limiting the efficacy of T cell directed therapies in solid tumors are presented in Fig. 1a.

### Challenges faced by both CAR-T cells and BiTEs

#### Tumor antigen specificity

As previously discussed, many tumor antigens found in solid tumors lack perfect specificity and are often found at low levels in normal tissue. An elegant demonstration of “on-target, off-tumor” toxicity was the development of CAR-T cells targeting carboxy-anhydrase-IX (CA IX) [10]. CA IX is expressed by many renal cell carcinomas, but low expression can also be found in normal tissue, including bile duct. In the phase 1 trial of CAR-T cells targeting CA IX, Lamers et al.reported frequent high-grade liver toxicities. They subsequently administered CA IX antibodies before CAR-T cell infusion which prevented this toxicity. However, no response was seen, and one might question the consequences of this strategy on efficacy [11].

Similar limitations have also been seen with BiTEs such as Solitomab (MT110, AMG 110), a bispecific antibody targeting EpCAM (an antigen frequently overexpressed in solid tumors, but also expressed at lower levels in normal tissue, notably gastrointestinal tract). The phase 1 could not determine an adequate dose, due to the occurrence of dose-limiting toxicities (DLTs), mostly transaminitis and diarrhea [12].

Some clinical trials testing CAR-T cells directed against CEA (CEACAM5) experienced lung toxicity [13]. Despite previous report suggesting no expression in normal lungs, the authors suggested that the CAR-T cells induced upregulation of CEA in the lung epithelium following infusion, due to cytokine production. They also found some expression of CEA in lung resection from non-cancer tissue, suggesting that the antigen is indeed present despite prior reports. Conversely, another product targeting CEA (autologous T cells engineered to express a murine TCR targeting CEA) was associated with severe colitis due to expression of CEA in the large intestine [14].

The first patient treated with a CAR-T cell targeting HER2 died of lung toxicity [8]. It was thought to be related to a low expression of HER2 by the lung, along with a severe cytokine release syndrome.

Lastly, CAR-T cells target only membranes protein. Intracellular antigens can only be targeted through natural or artificial TCR [15].

Thus, the selection of surface antigens for solid tumors may be hindered by limited expression of the antigen in normal tissue but sufficient to induce toxicity.

#### Tumor antigen heterogeneity

One of the most frequent escape mechanisms to CAR-T therapy in hematologic tumors is the loss of the target antigen [16]. Similarly, in patients treated with EGFRvIII-targeting CAR-T cells, 7 patients underwent surgery following treatment, due to clinical deterioration [17]. In 5 patients, decrease of EGFRvIII expression was confirmed; and one subject was found to have heterogeneous expression of EGFRvIII between different regions of the tumor. This analysis clearly demonstrated that solid tumors also frequently escape under the pressure of CAR-T cells, and that heterogeneous expression of the target might limit the efficacy of the product. A similar down-regulation of the target has also been reported in one patient who underwent surgery after treatment with IL13Ra2-targeting CAR-T cells for glioblastoma [18].

Similar mechanisms are likely to occur with BiTEs, and has been documented in hematologic malignancies with blinatumomab, a CD19/CD3 bispecific antibody [19].

#### Local immune suppression

In solid tumors, tumor infiltrating T cells may be rendered dysfunctional due to immunosuppressive mechanisms in the tumor microenvironment. These mechanisms include extrinsic suppression by regulatory cell populations, inhibition by ligands such as programmed death ligand-1 (PD-L1), metabolic dysregulation by enzymes such as indoleamine-2,3-dioxygenase, and the action of soluble inhibitory factors such as transforming growth factor-beta (reviewed in [20]). In preclinical models, CAR-T cells were found to have impaired functionality after reaching the tumor microenvironment [21]. In patients with glioblastoma treated with CAR-T cells targeting EGFRvIII, in situ evaluation of the tumor environment demonstrated increased expression of inhibitory molecules and infiltration by regulatory T cells [17].

### Challenges faced only by CAR-T cells or BiTEs

#### CAR-T cell manufacturing

Ex vivo engineering of CAR-T cells is currently complex from a logistical point of view. Viral vectors (either lenti- or retroviruses) are used for the manufacturing [22], in a process that is time-consuming and requires specialized biosafety level 2 facilities and trained staff resources. These manufacturing issues hinder their availability in clinical routine, at least for autologous CAR-T cells (Table 1). Production failures of CAR-T cells have been observed in a small percentage of patients with hematologic malignancies. The possibility to generate and expand CAR-T cells from patients with solid tumors who have been previously exposed to chemotherapy, and the rate of production failure are yet undetermined due to limited experience in these patients.

#### Exposure to the treatment

Exposure to the treatment represent different challenges for CAR-T cells and BiTEs.

CAR-T cells are injected after a lymphodepleting chemotherapy to facilitate their expansion and persistence in vivo. In hematologic malignancies, this expansion and persistence have been shown to correlate with efficacy. This might be related to the fact that anti-CD19 CAR-T cells directly encountered their target in the blood and could thus be immediately activated. In solid tumors, the expansion of CAR-T cells seems to be reduced. Expansion of anti-CEA CAR-T cells was shown to be limited [13]. Anti-HER2 CAR-T cells tested in the treatment of sarcomas persisted poorly with only low-levels detected at 6 weeks, and, at 3 months, only 4 out of 12 patients had still detectable CAR-T cells [23]. Short persistence was also an issue in 2 trials using CAR-T cells targeting TAG72 for the treatment of colorectal metastases [24]. CAR-T cells could only be detected during a short period (≤ 14 weeks). A similar observation was made with CAR-T cells targeting GD2 for the treatment of neuroblastoma [25]. There was no persistence even at higher doses and no CAR-T cells could be detected beyond Day45.

Low exposure to BiTEs was mainly due to the short half-life of many constructs (scFv), including first-generation BiTEs (half-life = 2 to 3 h for blinatumomab). This short half-life required continuous infusion. Discontinuous administration of a BiTE targeting CEA has been associated with low exposure, which may explain the lack of clinical activity in the phase I trial [26]. The administration was also associated with the development of anti-drug antibodies, which is also another cause of low exposure to drugs [27].

Moreover, T cell dysfunction is a hallmark of many cancer [28] and may negatively impact the results of both T cell-directed therapies. Albeit lack of clinical evidence, this should be an area of vigilance.

#### Trafficking to the tumors

In a phase I study of folate-receptor targeting cells, no specific trafficking to the tumors was seen, likely explaining the lack of clinical activity [29]. Another important finding of the CAR-T cells targeting TAG72 trials was that even if the cells were able to traffic to the tumors, they seemed to be excluded from the center of the tumor mass [24]. Trafficking through the blood–brain barrier might also be challenging although responses have been seen in hematologic malignancies with central nervous system (CNS) involvement [30].

While BiTEs do not actively traffic to the tumor, their activity is based on trafficking of endogenous T cells that can encounter similar difficulties. Their ability to penetrate in a tumor (for example through the blood–brain barrier, or in a hypovascular solid tumor) should also be confirmed.

## Opportunities to overcome the challenges

The different opportunities to overcome the challenges for T cell-directed therapies efficacy in solid tumors are presented in Fig. 1b.

### Improving antigen targeting

#### Selection of antigens with limited expression on normal tissue

Glypican-3 is a protein expressed in hepatocellular carcinoma with very limited expression on normal cells, and different trials have shown the feasibility and lack of toxicity of CAR-T cells targeting Glypican-3 [31]. A BiTE targeting Glypican-3 has also been developed [32]. Similarly, PSMA seems a promising target for T cell-directed therapies. In addition to a CAR-T cell trial [33], positive results of the BiTE pasotuxizumab were presented, showing decrease of PSA by more than 50% in 3 patients out of 15 treated, with many other patients experiencing lower decrease [34].

#### Discovery of novel antigens

Proteomics approaches have been used to discover new tumor-associated antigens with better specificity [35]. The use of antigens for which circulating T cells will have limited access in normal cells may be the source of less toxic therapies. CAR-T cell targeting GUCY2C, a glycoprotein expressed in normal tissue only on the luminal membranes but homogeneously on cancer cells, have been engineered to treat colorectal cancers [36]. Claudin 18.2 is a component of the tight junction, present only in gastric normal mucosa, and expressed in various solid tumors. Targeting of Claudin 18.2 by CAR-T cells has shown promising results [37], and a BiTE compound, AMG 910, has been developed and has now entered clinical evaluation [38, 39].

Finally, an interesting approach was to use chlorotoxin, a peptide known to bind specifically to glioblastoma cells, even if the binding site is not well defined. CAR-T cells targeting chlorotoxin have been developed in a preclinical model to treat glioblastoma [40].

#### Transient expression and safety switch

To mitigate toxicity, T cells with transient expression of the CAR have been designed. This would limit the off-target toxicity, but with the caveat of requiring repeat infusion of the cells. This approach was proved feasible in a trial of CAR-T cells targeting mesothelin for the treatment of pancreatic adenocarcinoma, with no toxicity reported, and stabilization in 2 out of 6 patients treated [41]. Another mechanism is the use of transcriptional activation in response to recognition of user-specified antigens, using synthetic Notch (synNotch) receptors [42, 43]. This allows the activation of the CAR-T cells only in the context of the tumor. Other approaches include the inclusion in the construct of a safety switch which enables removal of CAR-T cells by inducing their apoptosis in case they become too toxic [44–46].

#### Targeting intracellular antigens through TCR-directed therapies

Another important avenue is the use of TCR-directed therapies. One major advantage is to expand potential targeting to intracellular molecules, some of which can be found mutated in solid tumors. One caveat of the approach being that this kind of therapies will depend on MHC presentation and will thus be HLA-compatible. MHC expression downregulation by the tumor cells is a frequent immune escape mechanism and has also to be considered. Approaches similar to CAR-T cells have been used, in which a modified TCR is expressed, targeting the antigen of interest [47]. These types of construct has already been successful in targeting NY-ESO-1 for the treatment of multiple myeloma and synovial sarcoma [6, 48]. Frequent responses were seen, including in synovial sarcoma, with a similar product targeting MAGE-A4 [49]. Two different products targeting alpha-feto-protein, an antigen frequently expressed in hepatocellular carcinoma, have also been associated with responses [50, 51].

Similar strategies are pursed with BiTEs. The immune-mobilizing monoclonal TCRs against cancer (ImmTACs) target TCR-presenting antigens, and recruits effector cells [52]. A proof of concept of this approach was given with tebentafusp, a TCR/Anti-CD3 bispecific fusion protein targeting gp100, which was able to induce responses in melanoma patients, including difficult to treat uveal melanoma [53].

#### Using tumor-infiltrating lymphocytes

Another avenue for ACT in solid tumors consists in the use of endogenous T cells. TILs can be harvested from the tumor, then expanded in vitro, and finally infused back into the patients. This way, efficacy might be found despite ignoring the actual target of the infused T cells. Positive results were shown in several types of tumors [54–59].

Thus, many new options are used to develop alternative ways to target tumor cells.

### Combinational approaches to tackle tumor heterogeneity

Tumor heterogeneity could be bypassed by eliciting immunogenicity towards a broader range of antigens. This is the concept of epitope spreading, where the CAR-T cells express co-stimulatory molecules such as CD40L or 4-1BBL to stimulate endogenous immune responses [60, 61].

Antigen loss and tumor heterogeneity were shown to be an important escape mechanism to T cell-directed therapies. Targeting different tumor antigens is a theoretical approach to tackle tumor heterogeneity. Bicistronics CAR-T cells targeting both CD19 and CD22 are being developed in the clinic [62, 63]. The same group described the first tandem CAR-T cells targeting HER2 and IL13Ra2, then trivalent CAR-T cells targeting HER2, IL13Rα2 and ephrin-A2, and showed better results in preclinical models of glioblastoma compared to monovalent CAR-T cells [64, 65]. Another similar approach is to treat a patient with different CAR-T cells targeting different antigens [66].

An elegant approach has been used to overcome EGFRvIII antigen loss, with EGFRvIII-targeting CAR-T cells which secrete a BiTE targeting wild-type EGFR [67]. With this product, CAR-T cells are directed to the tumor due to the specificity of the EGFRvIII mutation, secrete a BiTE that can target tumor cells expressing normal EGFR through tumor heterogeneity or antigen loss. The expression by the CAR-T cells avoid systemic exposure to the EGFR BiTE which would have resulted in “on-target, off-tumor” toxicity. This is also a fine example of how CAR-T cells and BiTEs can be combined.

### Improving exposure, persistence and activity of the products

#### Extending BiTEs half-life

New half-life extended BiTEs have been developed to improve the exposure of BiTEs, and to avoid continuous infusion [68]. Initial BiTEs lack the Fc portion, and thus were not enable to recycle through neonatal crystallizable fragment receptor-mediated (FcRn). Fusion with the Fc domain enables engineering of new BiTEs with extended half-life and discontinued administration.

#### Improving BiTEs activity

A further development is the use of trispecific antibodies (TriTE), targeting one antigen but using different T-cell engagement molecules. One example is the anti-myeloma compound SAR442257 which targets CD38, and recruits T cells via CD3 and CD28 [69, 70]. Efforts are currently conducted to develop such molecules for the treatment of solid tumors, but none have reached clinical stage yet [71].

#### Improving persistence and tackling exhaustion of CAR-T cells

Persistence and activity of CAR-T cells were improved in second- and third-generation CAR-T cells, where the products also contain additional costimulatory domains (4-1BB, CD28, OX40 and/or ICOS) [2, 72]. It is also important to use an adequate lymphodepleting regimen, which has been shown to improve results of HER2-targeting CAR-T cells for the treatment of sarcoma [73, 74]. Moreover, it was shown that different types of T cells might have different killing potency, the naïve and central memory T cells having higher activity than effector memory T cells, suggesting that the selection of a correct proportion of the different cells might improve the overall results [75]. This process might be automated [76]. A deep analysis of a responding patient showed that a single clone, in which the CAR was inserted randomly within the *TET2* gene, was responsible for the response [77]. The disruption of *TET2* induced a central memory phenotype, which was probably responsible for its increased activity. While modification of *TET2* might be too risky, modifications of other parameters related to exhaustion, such as *NR4a* or *Jun* might augment CAR-T activity [78, 79].

### Improving trafficking

The delivery of the drug to the tumor site might be improved by different ways. A platform to enable bispecific biologics to cross the blood–brain barrier has been developed [80].

For CAR-T cells, multiple trials investigated local injection. This could be done in an anatomical cavity (pleura, peritoneum), via a device placed surgically (for CNS tumors) or via intra-arterial delivery (such as hepatic artery catheterization), or by direct intra-tumoral injection. The described response of a glioblastoma to IL13Rα2-targeted CAR-T was achieved after intracranial delivery [81]. Intra-tumoral injection of cMET targeting CAR-T cells was shown to be feasible in metastatic breast cancer [82]. Feasibility of intra-pleural injection of CAR-T cells targeting mesothelin was also demonstrated [83]. Intra-arterial hepatic infusion of CEA-targeting CAR-T cell was done in different clinical trials, with one patient showing response [84, 85].

Another strategy to improve trafficking is to make the CAR-T cells express chemokine receptors that can redirect the CAR-T cells into the tumors. CCR2-expressing CAR-T cells targeting GD2 were shown to have better activity in vivo [86]. There is however controversy about the best chemokine receptor to use for improved trafficking [87].

### Reversing the immunosuppressive microenvironment

#### Combination with immune checkpoint inhibitors

With the advent of ICI, their use in combination with CAR-T cells and BiTEs become an evident avenue of research. First evidence of potential additive effects was shown in a phase 1 trial of a BiTE targeting CEA in patients with metastatic colorectal cancer [88]. The trial had 2 cohorts, with or without the addition of the anti-PD-L1 antibody atezolizumab. The response rates without and with atezolizumab were 6% vs 18% (only 1 patient was MSI, all other responders were MSS) respectively, while the disease control rates were 45% vs 82%, respectively. Combination trials of CAR-T cells and ICI have also been presented, with a suggestion that ICI improved responses in patients treated with intra-pleural infusion of mesothelin-targeting CAR-T cells [83]. Conversely, a small phase 1 trial of combinations based on GD2 CAR-T cells showed that the lymphodepletion regimen had huge impact, but the addition of the anti-PD-1 antibody pembrolizumab did not clearly improve the results (but the number of patients was very limited and the follow-up was short) [89]. Several clinical trials are ongoing using such combination. A derived approach is to develop BiTE targeting PD-L1 expressing cells (so called checkpoint-inhibitory T cell engagers (CiTEs)) that will target the cells promoting immunosuppression [90].

#### Expression of cytokines

Another approach to tackle the immunosuppressive microenvironment is to make the CAR-T cells secrete stimulatory cytokines. In a preclinical model of pancreatic cancer, IL-18 expressing CAR-T cells targeting CEA improved results over conventional CAR-T cells [91].

Finally, some authors proposed the combination of local treatment with CAR-T cell administration. For example, radiation was suggested to have the potential to improve Immuno-Oncology results, and combination of intra-arterial hepatic infusion of CEA-targeting CAR-T cells with selective internal radiation therapy was demonstrated feasible and associated with responses in liver metastases [92].

### Improving CAR-T cell manufacturing

Autologous CAR-T cells are patient-derived personalized products, thus associated with the absence of allogeneic rejection, which enables long-term persistence. Nonetheless, this bespoke manufacturing process presents many disadvantages, such as a delay in the availability of the treatment (2 to 4 weeks), a complex manufacturing procedure and an increase cost. Current CAR-T cell engineering mainly uses viral vectors, that should be handled in specialized facilities. Alternative insertion strategies are under development, such as sleeping beauty or piggy-bac transposons, that could facilitate the manufacturing processes [22]. Another strategy to potentially address these issues is the development of universal CAR-T cells [93]. The principle is to use allogeneic T cells from healthy donors in order to produce “off-the-shelf” allogeneic CAR-T cells. This approach has the potential to simplify and scale-up the manufacturing processes, allowing for an immediate delivery of the treatment at reduced costs [94]. In order to produce allogeneic CAR-T cells with no potential for graft-versus-host disease (GVHD), the endogenous TCR should be disrupted through gene editing (zinc finger nuclease, transcription activator-like effector nuclease (TALEN) or CRISPR/Cas9 methods) [22, 95]. However, allogeneic CAR-T cells may be rapidly eliminated by the host immune system, thus limiting their persistence and clinical efficacy. Application of TALEN-based universal CAR-T cells targeting CD19 (UCART19) was recently reported for 21 patients with relapsed/refractory B cell acute lymphoblastic leukemia [96]. Despite high rates of complete remission among patients who received the most immunosuppressive conditioning regimen, a short duration of response and limited CAR-T persistence were observed, and a majority of patients had to undergo allogeneic stem cell transplantation. These results emphasize the need to decrease allogeneic rejection of universal CAR-cells. Regarding solid tumors, a preclinical model of universal EGFRvIII CAR-T cells has been developed [97]. Phase 1 clinical trials with universal CAR-T cells targeting mesothelin (NCT03545815) and NKG2D (NCT03692429) are ongoing.

## Conclusion and perspectives

CAR-T cells and BiTEs both represent promising approaches of immunotherapy and are still at the beginning of their clinical development in solid tumors. The last generations of such T cell-directed therapies have the potential to overcome the challenges they are facing and have shown promising preclinical results. None can now predict how CAR-T cells and/or BiTEs will be used in the future treatment strategies and large clinical studies are eagerly awaited. At this point, it is difficult to speculate which cancers will benefit most from these therapies since clinical results in solid tumors are limited. One may expect that tumors which are more likely to benefit from CAR-T cells and BiTEs are: i) tumors which do not respond to checkpoint inhibitors because they lack pre-existing antitumor T cells (“cold tumors”), ii) tumors with a targetable surface antigen (either a surface neoantigen or an overexpressed antigen which can be targeted with limited organ toxicity) which is expressed homogeneously by all tumor cells (to avoid immune escape), and iii) tumors with a permissive and poorly immunosuppressive microenvironment.

To date, the development of CAR-T cells and BiTEs has focused on chemo-refractory/relapsed patients. However, these immunotherapies may be more efficient if given earlier in the therapeutic strategy, as suggested in lymphoma patients [98]. In the future, these immunotherapies could also be combined with standard chemotherapy and/or targeted therapies: these treatments could reduce the tumor burden and/or modulate the immune response [99, 100].

Finally, the question may be not to compare CARs *versus* BiTEs, or how these innovative immunotherapies will compare to standard chemotherapy, but whether how to combine all therapeutic modalities.

## Data Availability

Not applicable.

## Abbreviations

Ab: Antibody
ACT: Adoptive cell therapy
Ag: Antigen
BiTE: Bispecific T cell engager
CA IX: Carboxy-anhydrase-IX
CAR: Chimeric antigen receptor
CiTEs: Checkpoint-inhibitory T cell engagers
CNS: Central nervous system
DLTs: Dose-limiting toxicities
FcRn: Neonatal fragment crystallizable receptor
GVHD: Graft-versus-host disease
ICI: Immune checkpoint inhibitors
MHC: Major histocompatibility complex
PD-L1: Programmed death ligand-1
scFv: Single-chain variable fragment
TALEN: Transcription activator-like effector nuclease
TCR: T cell receptor
TILs: Tumor-infiltrating lymphocytes
TriTE: Trispecific antibodies
